# Effects of 8 Weeks of Neuromuscular and SAQ Training on Physical Performance in Youth Soccer Players

**DOI:** 10.3390/jcm15031202

**Published:** 2026-02-03

**Authors:** Yu-Bin Lee, Kwang-Jin Lee, Se-Young Seon, Keun-Ok An

**Affiliations:** 1 Department of Sports Medicine, Korea National University of Transportation, Chungju-si 27469, Republic of Korea; lkv5596@naver.com; 2 Department of Exercise Prescription, Jeonju University, Jeonju-si 55069, Republic of Korea; rhkdwls@jj.ac.kr

**Keywords:** youth soccer players, neuromuscular, speed, agility, quickness

## Abstract

**Backgrounds/Objectives:** Adolescent soccer players are exposed to elevated injury risk due to rapid musculoskeletal development and high physical demands. Neuromuscular training (NMT) and speed–agility–quickness (SAQ) training are widely used to enhance performance and reduce injury risk in youth athletes. While both approaches are effective, comparative evidence regarding their modality-specific performance adaptations remains limited. Furthermore, few studies have discussed how such performance data may inform evidence-based or data-driven training selection in youth sports contexts. **Methods:** Thirty-six male youth soccer players with at least three years of playing experience, affiliated with Team A in Gyeonggi-do and Team B in Chungcheongbuk-do, participated in the study (NMTG, *n* = 21; SAQG, *n* = 15). Participants completed either an NMT or SAQ training program for eight weeks. To objectively assess exercise performance, pre- and post-tests were conducted measuring dynamic balance, vertical jump, zigzag run, and carioca. **Results**: Findings revealed a significant main effect of time for lower limb power (*p* < 0.05), but no significant group × time interaction, indicating that both NMTG and SAQG improved significantly over the 8-week period. Conversely, significant interaction effects were found for agility (*p* < 0.001), with SAQG demonstrating superior enhancements compared to NMTG. Dynamic balance showed no significant time effect or interaction. **Conclusions:** While NMTG and SAQG are equally effective for enhancing lower limb power, SAQG provides modality-specific advantages for agility in youth soccer players. These results emphasize time-dependent adaptations for power and the distinct benefits of SAQG for multi-directional speed. These adaptation profiles offer a data-driven framework for optimizing training selection in youth athletes.

## 1. Introduction

Youth athletes exhibit incomplete development of muscles, tendons, ligaments, and joints [1]. In particular, soccer involves repetitive high-intensity activities—such as rapid changes in direction, acceleration, deceleration, and jumping—highlighting the critical need for targeted training to supplement and support these physical demands [2,3]. Optimizing physical performance and ensuring sustainable athletic development during this critical growth period are fundamental to the long-term career progression of youth soccer players [4].

It has been reported that youth soccer players in their growth phase with higher levels of exercise performance tend to have lower injury rates during matches, and that the appropriate implementation of injury prevention programs can significantly reduce the risk of injuries [5].

Recently, neuromuscular training (NMT) programs have gained popularity as an effective approach to enhance exercise performance and prevent injuries among youth soccer players [6]. NMT refers to exercise programs that integrate specific biomechanical components such as resistance, dynamic stability, balance, core strength, plyometrics, and agility, with the aim of improving performance and minimizing injury risk [7]. Such neuromuscular interventions are primarily designed to address intrinsic risk factors associated with sports injuries [8,9].

Previous studies on neuromuscular training (NMT) have demonstrated its effectiveness in both injury prevention and performance enhancement. For example, Trajković et al. (2020) reported that the application of the FIFA 11+ program, widely recognized for its neuromuscular control benefits, led to significant improvements in jump performance and agility [10]. In a broader context, Myer et al. (2013) emphasized that structured NMT programs incorporating dynamic stability, balance, and plyometric exercises significantly reduced the incidence of lower extremity injuries and enhanced functional movement patterns in young athletes [11]. Furthermore, Steffen et al. (2013) suggested that improvements in dynamic balance observed after participating in the FIFA 11+ program may contribute to a reduced risk of sports injuries [12].

On the other hand, Speed, Agility, and Quickness (SAQ) training is widely implemented to maximize athletic performance by targeting explosive speed and multi-directional movement efficiency [13,14]. This systematic approach is designed to improve acceleration and change-of-direction (COD) ability [13]. Through SAQ training, youth soccer players can develop faster movement patterns and more refined motor control during high-velocity maneuvers, which are essential for success in competitive match play [15,16].

Although both NMT and SAQ training aim to enhance movement quality, they are distinct in their primary objectives: NMT focuses on enhancing neuromuscular control and movement efficiency through core stability and balance, whereas SAQ emphasizes maximizing explosive acceleration and multi-directional speed. Despite these conceptual differences, there is a lack of comparative research examining the distinct adaptation profiles of these two training approaches in youth soccer athletes. Investigating these differences is crucial for developing data-driven training selection frameworks tailored to the specific athletic needs of youth players.

Therefore, the purpose of this study is to investigate and compare the effects of an 8-week NMT program and an SAQ training program on various aspects of physical performance in youth soccer players. We hypothesized that: First, both NMT and SAQ training would significantly improve physical performance markers compared to baseline. Second, SAQ training would be superior to NMT in enhancing multi-directional agility. Third, NMT would lead to more pronounced improvements in lower limb power and stability-related performance.

## 2. Methods

### 2.1. Participants

Participants were selected from 36 male youth soccer players from soccer team A in Gyeonggi-do and soccer team B in Chungcheongbuk-do who had at least 3 years of soccer experience at the time of the study (Table 1). Subjects were selected if they had attended soccer training for 6 months, had no musculoskeletal disorders within 6 months, and had not participated in structured resistance training in the past year.

Biological maturation was estimated using the maturity offset equation [17]. Maturity offset (years) = −7.999994 + (0.0036124 × [Age × Height]). At baseline, there were no significant differences in maturity offset between the NMTG and SAQG (*p* > 0.05), ensuring group equivalence regarding somatic maturation.

### 2.2. Experimental Design

**Figure 1:**
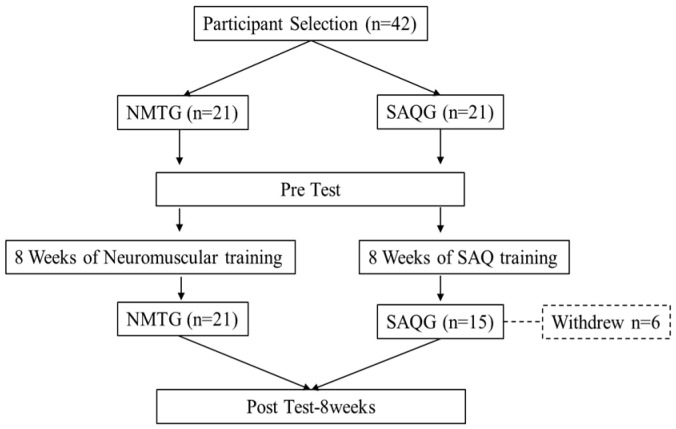
Experimental design.

This study employed a quasi-experimental design using two pre-existing youth soccer teams. Participants were allocated to the training groups based on their team affiliation to minimize disruption to training schedules: Team A was assigned to the Neuromuscular training group (NMTG, *n* = 21), and Team B was assigned to the Speed, Agility, Quickness Training group (SAQG, *n* = 21). Detailed information on the purpose of the study, the content of the test, the effect of exercise, and the exercise method was provided, and the data of a total of 36 participants, 21 NMTG and 15 SAQG, were analyzed after excluding subjects who could not complete the post-measurement due to injury during the study. This study was approved after deliberation by the Institutional Review Board (IRB) of Korea National University of Transportation (KNUT IRB 2023-02), and all evaluations were conducted before and after 8 weeks of exercise intervention (Figure 1).

### 2.3. Measuring Physical Performance

1) Dynamic Balance Test.

**Figure 2:**
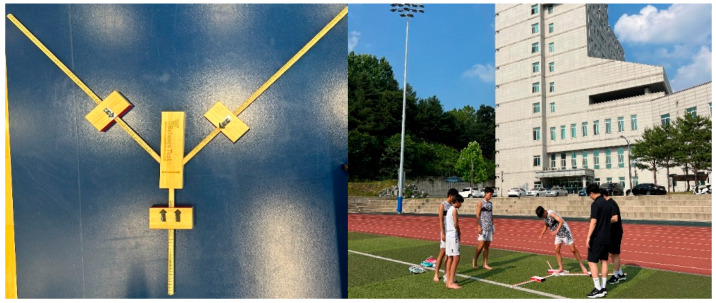
Y-Balance test.

Dynamic balance ability was assessed using the Y-Balance Test device. Participants stood barefoot on the central fixed platform with the stance (supporting) limb, while reaching as far as possible in the anterior, posteromedial, and posterolateral directions by extending the contralateral (non-supporting) lower limb [18]. Each direction was measured twice, and the highest value was recorded. Trials were repeated after a 3 min rest if the marker was displaced, the reaching leg touched the floor, or balance was lost during the attempt. A composite score was calculated based on the recorded values. Lower limb length was measured from the anterior superior iliac spine (ASIS) to the medial malleolus. The composite score was calculated using the formula: Composite Score = (ANT + PM + PL)/(3 × Lower Limb Length) (Figure 2) [19,20].

2) Power Test.

**Figure 3:**
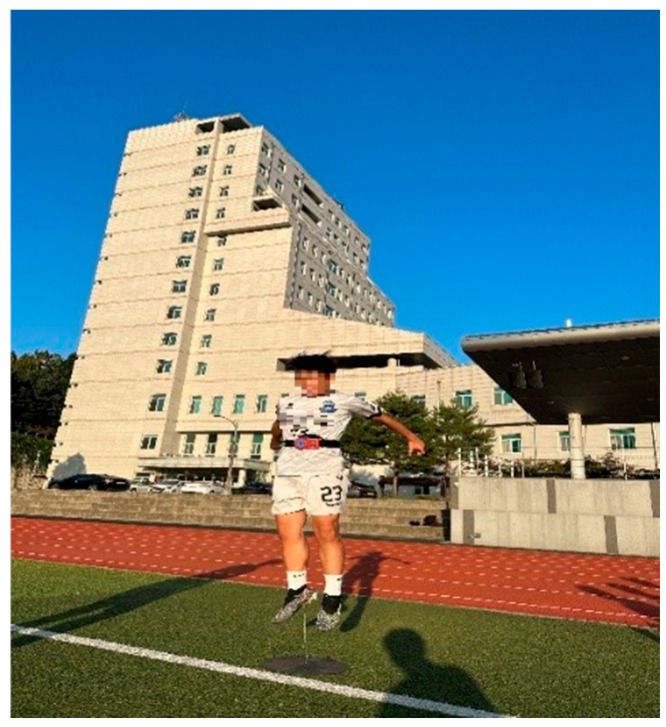
Countermovement jump.

Power was measured through the countermovement jump. The vertical jump measuring device (T.K.K. 5414, Takei Scientific Instruments Co., Ltd., Niigata, Japan) was used to measure the jump distance. The highest value of two starts was used, and a 1 min rest period was given between repetitions of the same jump motion. After quickly reaching the knee angle of 90° as suggested by Kozinc et al., the hip, knee, and ankle were quickly extended as an immediate action to jump as high as possible (Figure 3) [21].

3) Agility Test.

**Figure 4:**
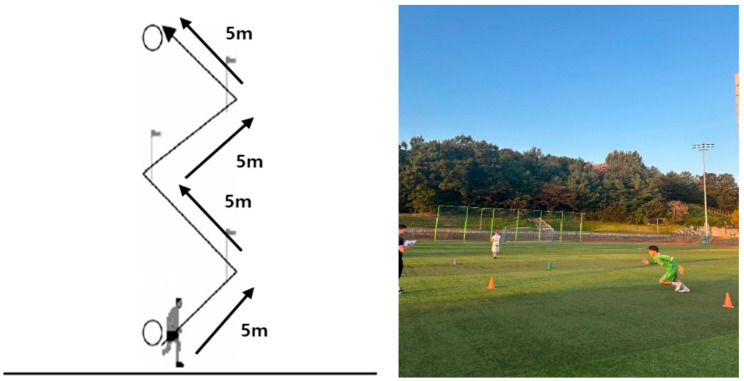
Zigzag run test.

Agility was measured using the Zigzag run test and Carioca. Each subject was measured twice, and the fastest record was used. The rest time for each repetition of the measured movement was 2 min, and the rest time between the measured items was 3 min. Details of each measurement item are as follows. The Zigzag Run Test, designed to assess agility, was performed by placing four markers at 5 m intervals from the starting line, with each marker set at a 100° angle relative to the previous one. From the starting position, participants sprinted toward each marker in sequence, changing direction at each point, and continued sprinting until reaching the final marker and the designated finish line (Figure 4) [22].

**Figure 5:**
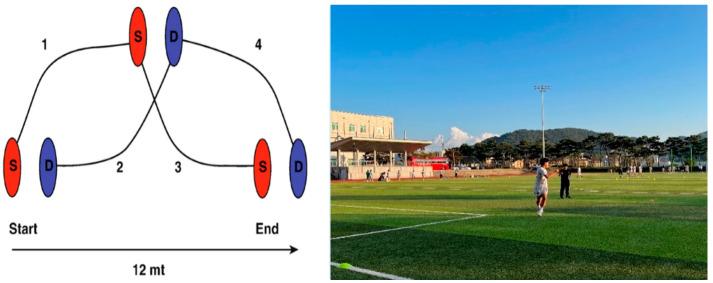
Carioca test.

Table Carioca Test, designed to assess agility and coordination, was performed twice, and the fastest time (i.e., the lower of the two trials) was recorded. A 2 min rest period was provided between trials. In this test, participants performed lateral movement using crossover steps—alternately crossing one foot in front of and behind the other—while moving sideways. The time required to cover a 12 m distance from the starting line to the finish line was measured using a stop-watch (Figure 5) [23].

The reliability of the selected tests has been well-established. Reported Intraclass Correlation Coefficients (ICC) for these protocols are as follows: 0.85–0.91 for the Y-Balance Test [18], 0.98 for the CMJ [24], 0.96 for the Carioca test [25], and 0.82–0.94 for the Zigzag run test [26].

### 2.4. Exercise Intervention Program

1) Neuromuscular Training Program

The neuromuscular training program was designed to enhance neuromuscular control, coordination, and movement efficiency by integrating specific biomechanical components such as resistance, dynamic stability, balance, core strength, and plyometric exercises. Unlike traditional strength training, this program focused on optimizing neuromuscular pathways and movement quality, which are essential for the physical demands of youth soccer [27,28,29]. The program was tailored to the developmental characteristics of adolescent players to ensure age-appropriate progression. Each training session lasted 30 min and was conducted three times per week over an 8-week period. Prior to the intervention, participants received a two-day orientation to ensure proper execution of the exercises. Training intensity was progressively adjusted each week by modifying volume, type, speed, and sets (Table 2).

2) SAQ Training Program

The SAQ (Speed, Agility, and Quickness) training program was designed to enhance the biomechanical elements required in soccer by integrating speed, agility, and quickness-based physical conditioning. The program was developed with reference to previous studies and adapted to suit the developmental characteristics of youth soccer players [30,31]. Each training session lasted 30 min and was performed three times per week over an 8-week period. A two-day instructional period was provided prior to the intervention to ensure accurate execution of movement patterns. Training intensity was progressively adjusted each week by modifying exercise volume, type, speed, and number of sets (Table 3).

### 2.5. Data Analysis

All statistical analyses were performed using IBM SPSS Statistics for Windows, Version 27.0 (IBM Corp., Armonk, NY, USA). Descriptive statistics are presented as mean ± standard deviation (M ± SD). To evaluate the effects of the intervention, a two-way repeated measures analysis of variance (ANOVA) was performed to examine the interaction between group (NMTG vs. SAQG) and time (pre- vs. 8-week post-intervention). To control for the inflation of Type I error due to multiple comparisons, Bonferroni adjustments were applied to all post hoc pairwise comparisons. Simple main effect analyses were conducted only when a significant Group x Time interaction was observed. For variables without a significant interaction, the interpretation was strictly focused on the main effect of time. Effect sizes were reported using partial eta squared ($n_{p}^{2}$) for the ANOVA effects and Cohen’s *d* for within-group changes to indicate the magnitude of the intervention effects. The level of statistical significance was set at *p* < 0.05.

## 3. Results

### 3.1. Dynamic Balance Test

The statistical analysis of dynamic balance ability, assessed via the Y-Balance Test, revealed a significant main effect of group for both the left foot (*F* = 12.858, *p* < 0.001, $n_{p}^{2}$ = 0.274), and the right foot (*F* = 14.218, *p* < 0.001, $n_{p}^{2}$ = 0.295) indicating consistent differences in balance performance between NMTG and SAQG.

**Figure 6:**
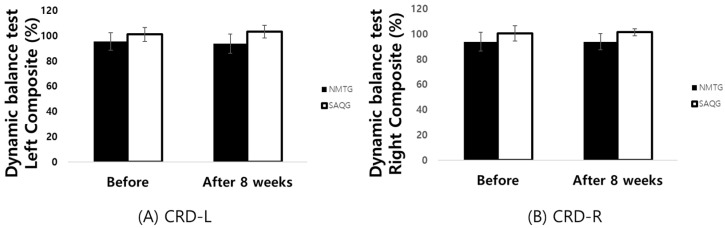
(**A**) CRD-L, composite reach distance-left. (**B**) CRD-R, composite reach distance-right.

However, no significant main effect of time was observed for either the left (*F* = 0.119, *p* < 0.732, $n_{p}^{2}$ = 0.003) or right foot (*F* = 0.319, *p* < 0.576, $n_{p}^{2}$ = 0.009). Furthermore, there was no significant interaction effect between group and time (Left: *F* = 7.086, *p* < 0.120, $n_{p}^{2}$ = 0.172; Right: *F* = 0.325, *p* < 0.572, $n_{p}^{2}$ = 0.009), suggesting that neither training modality led to statistically significant changes in dynamic balance over the 8-week period (Table 4; Figure 6).

### 3.2. Power Test

**Figure 7:**
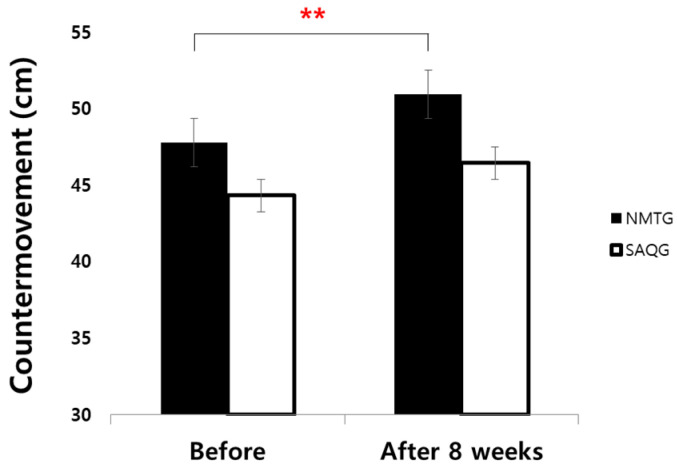
Change in Countermovement jump. ** *p* < 0.01.

Regarding the countermovement jump (CMJ), which measures lower limb power, there was no significant main effect of group (*F* = 3.79, *p* < 0.060, $n_{p}^{2}$ = 0.100). However, a significant main effect of time was identified (*F* = 10.864, *p* < 0.01, $n_{p}^{2}$ = 0.242), indicating an overall improvement in performance across the measurement periods. No significant interaction effect between group and time was observed (*F* = 0.398, *p* < 0.532, $n_{p}^{2}$ = 0.012). In accordance with the statistical outcomes, these results suggest that both NMTG and SAQG experienced significant enhancements in lower limb power over the 8-week intervention, and these changes cannot be conclusively attributed to one training modality over the other (Table 5; Figure 7).

### 3.3. Agility Test

**Figure 8:**
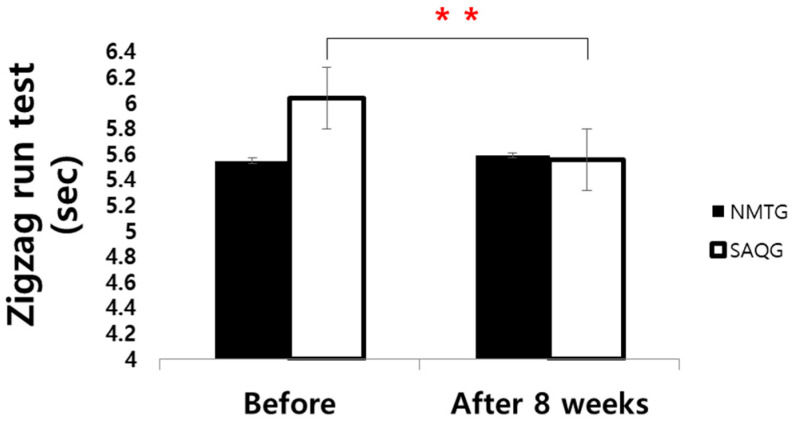
Change in Zigzag run test. ** *p* < 0.01.

Zigzag Run Test For the Zigzag Run, significant main effects were found for group (*F* = 2.573, *p* < 0.001, $n_{p}^{2}$ = 0.070), time (*F* = 14.069, *p* < 0.001, $n_{p}^{2}$ = 0.293), and interaction (*F* = 20.268, *p* < 0.001, $n_{p}^{2}$ = 0.373). Post hoc analysis revealed that significant improvement occurred specifically in the SAQG (*p* < 0.001, *d* = 0.75), while no significant change was observed in the NMTG (*p* = 0.213, *d* = 0.18) (Table 6; Figure 8).

**Figure 9:**
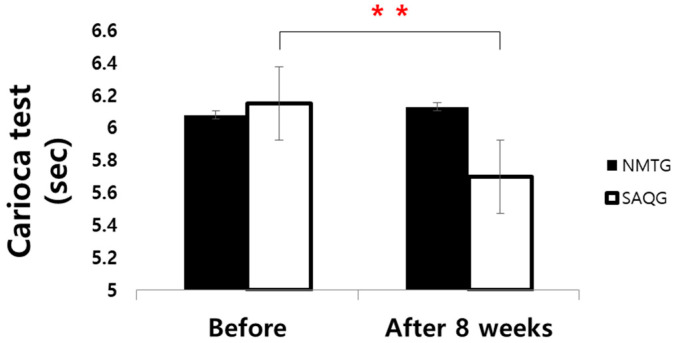
Change in Carioca test. ** *p* < 0.01.

Regarding the Carioca test, no significant main effect of group was observed (*F* = 1.355, *p* < 0.252, $n_{p}^{2}$ = 0.038). However, there was a significant main effect of time (*F* = 10.923, *p* < 0.002, $n_{p}^{2}$ = 0.243) and a significant group x time interaction (*F* = 18.174, *p* < 0.001, $n_{p}^{2}$ = 0.348). Post hoc analysis confirmed a significant enhancement specifically within the SAQG (*p* < 0.001, *d* = 0.78), whereas the NMTG demonstrated no significant alteration in performance over the 8-week period (*p* = 0.332, *d* = 0.12) (Table 7; Figure 9).

## 4. Discussion

This study aimed to compare the effects of an 8-week Neuromuscular Training (NMT) program and a Speed, Agility, and Quickness (SAQ) training program on dynamic balance, lower limb power, and agility in youth soccer players. The primary findings indicate that the Neuromuscular Training Group (NMTG) demonstrated improvements in lower limb power, whereas the SAQ Training Group (SAQG) showed significant improvements in agility. No within-group effects were observed for dynamic balance, although an interaction effect between group and time was found.

### 4.1. Dynamic Balance

In terms of dynamic balance, a two-way analysis of variance (Two-Way ANOVA) revealed a significant interaction effect between time and group. However, paired *t*-tests within each group showed no statistically significant changes over time in either the NMTG or the SAQG. This result contrasts with previous findings by Granacher et al. (2010) and Zhang et al. (2021), who reported that neuromuscular training could positively affect balance in adolescents [32,33]. The observed interaction effect suggests differing trends between groups, but the lack of significant within-group differences warrants cautious interpretation. The findings may be attributable to high baseline levels of balance ability among participants or insufficient intensity, frequency, or task specificity of the training protocols. Similarly, Cerrah et al. (2016) emphasized the need for long-term interventions to effectively improve balance in youth soccer players [34]. Furthermore, Nageswaran, A. S. (2013) noted that short-term SAQ interventions might provide transient improvements in balance; however, the 8-week duration in this study may have been insufficient to elicit sustained enhancements [35].

### 4.2. Power

Regarding vertical jump performance, a representative measure of lower limb power, a significant main effect of time was observed, indicating that both the NMTG and SAQG achieved significant improvements following the 8-week intervention. These findings are consistent with previous studies by Myer et al. (2006) and González-Fernández et al. (2024), which demonstrated that neuromuscular and strength-based training effectively enhance explosive lower limb force in youth athletes [36,37]. The improvements observed in both groups may be attributed to the rapid neuromuscular development characteristic of adolescence, where targeted interventions can effectively reduce neuromuscular fatigue and enhance movement control. While some previous literature, such as Jovanović et al. (2011), suggested that SAQ training primarily focuses on speed and agility rather than vertical power [31], our results indicate that the explosive acceleration and deceleration components inherent in SAQ drills also significantly contribute to vertical jump performance in youth soccer players. Consequently, both NMT and SAQ training appear to be effective modalities for optimizing lower limb power during this critical growth phase, with no statistically significant difference in efficacy between the two approaches.

### 4.3. Agility

In the current study, significant interaction effects were observed in agility performance, with the SAQG demonstrating superior gains compared to the NMTG. Specifically, significant improvements in the Zigzag Run Test were observed only in the SAQG. These results are highly consistent with Milanović et al. (2013), who reported that a structured SAQ program significantly enhances change-of-direction speed both with and without the ball in young soccer players [13]. Furthermore, our findings align with Anwer et al. (2021), who demonstrated that SAQ training, regardless of the use of specialized equipment, is more effective than traditional methods for improving high-velocity athletic performance parameters [38]. The superiority of SAQ training in the Zigzag Run can be attributed to the Principle of Specificity. The SAQ protocol involves repetitive, high-intensity directional changes that require rapid transitions between eccentric deceleration and concentric re-acceleration. This process optimizes motor unit recruitment and enhances the rate of force development (RFD), which are critical for the multi-directional agility required in competitive soccer scenarios. Regarding the Carioca Test, only the SAQG showed significant improvements over the 8-week period. This result is strongly supported by Alviana et al. (2020), who established that specific footwork drills, such as ladder drills and Carioca exercises, directly improve lateral speed and coordination [39]. The Carioca test necessitates sophisticated hip rotation control and lateral leg crossover patterns. The SAQ program utilized in this study included ladder-based drills and rapid lateral displacement tasks, which likely optimized the neural pathways responsible for pelvic stability and synchronized lower-limb movement [13,39]. From a developmental perspective, the high responsiveness of adolescent athletes to SAQ training suggests that high-velocity motor tasks are particularly effective in facilitating neuroplastic adaptations. By improving the coordination between the central nervous system and the musculoskeletal system, SAQ training allows youth players to overcome the temporary coordination deficits often seen during growth spurts—frequently termed ‘adolescent clumsiness’—thereby achieving more refined movement control during rapid changes in direction.

### 4.4. Clinical Implications

The findings of this study provide several critical clinical implications for sports medicine practitioners and pediatric clinicians. First, the integration of SAQ training offers a targeted intervention to mitigate ‘adolescent clumsiness’—a transient period of neuromuscular awkwardness during growth spurts that is often associated with a higher risk of non-contact musculoskeletal injuries [1]. By enhancing multi-directional agility and neuroplastic adaptations, SAQ training may serve as a preventive therapeutic modality for youth athletes. Second, the use of biological maturation data (Maturity Offset) for training prescription demonstrates a shift toward personalized pediatric sports medicine, allowing clinicians to tailor exercise loads based on a child’s physiological readiness rather than chronological age [17]. Lastly, the high-quality performance parameters established in this study provide a foundational dataset for future AI-driven diagnostic and predictive models for athletic injury risk, aligning with the evolving landscape of digital health and precision medicine in sports rehabilitation.

## 5. Limitations

This study has several limitations that should be considered when interpreting the results. First, participants were allocated to training groups based on team affiliation (Team A as NMTG and Team B as SAQG) rather than individual randomization due to logistical constraints in a field setting. Second, the sample size was relatively small (*n* = 36), and the study was restricted to male youth soccer players, which may limit the generalizability of the findings to female athletes or different age groups. Third, while somatic maturity status (e.g., maturity offset and PHV) was estimated using the Moore equation to characterize the participants, the narrow chronological age range may not fully capture the diverse biological maturation profiles that occur during the entire adolescent period. Fourth, this study compared two specific interventions without a passive control group, making it difficult to fully isolate the training effects from adaptations gained through regular soccer practice. Finally, although this study assessed performance-based proxies (balance, power, and agility) that are essential for long-term athletic development, it did not track actual injury incidence rates. Future research should include longitudinal follow-ups to determine whether these performance improvements translate into a reduced risk of lower extremity injuries during competitive play.

## 6. Conclusions

In conclusion, the 8-week intervention demonstrated that both Neuromuscular Training (NMT) and SAQ Training were effective in enhancing lower limb power, as evidenced by a significant main effect of time. However, SAQ Training produced superior, modality-specific improvements in agility, particularly in the Zigzag Run and Carioca Tests, where significant interaction effects were observed. Neither intervention led to substantial improvements in dynamic balance, possibly due to the participants’ high baseline performance or the 8-week duration being insufficient for balance-specific adaptations. From a performance optimization perspective, these findings suggest that SAQ training is a more effective strategy for enhancing multi-directional agility, while both NMT and SAQ can be utilized to improve explosive power in youth soccer players. This evidence supports the implementation of data-driven training selection to maximize the athletic potential of youth athletes during critical growth phases.

## Figures and Tables

**Table 1:** Physical Characteristics of the Subject.

Group (*n* = 36)	Age (yrs)	Height (cm)	Weight (kg)	BMI (kg/m^2^)	Maturity Offset (yrs)
NMTG (*n* = 21)	14.57 ± 0.50	167.97 ± 9.37	56.01 ± 14.43	20.40 ± 2.10	0.81 ± 0.67
SAQG (*n* = 15)	14.00 ± 0.92	168.70 ± 6.30	58.40 ± 9.99	20.40 ± 2.90	0.54 ± 0.82
*p*-value	0.061	0.797	0.586	0.992	0.285

Values are mean ± SD, NMTG, neuromuscular training group; SAQG, speed, agility, quickness training group; BMI, body mass index.

**Table 2:** Neuromuscular training program.

Time	Type		Intensity	Frequency
Warm-up (10 min)	Dynamic stretching			3 times per week for 8 weeks
Main exercise (30 min)	- Stability ball, Rocker board, and Bosu instrument Two leg → one leg (Lifting using ball)		5 m	
	- 1~2 weeks: Box jump, Drop landing MB throws - 3~4 weeks: CMJ, MB throws, ½ ankle jumps - 5~6 weeks: DJ (20 cm), MB Throws, Leg box hopping - 7~8 weeks: MB throws Lat. bounds + stabilization, Hurdle jumps (60 cm), DJ (40 cm)		2 sets × 6 reps 3 sets × 6 reps 3 sets × 8 reps 3 sets × 8 reps	
	- Squat - plank - Lunge & Side lunge - Single-Leg Rumanian Deadlift - Superman Dead bug	- 1~2 weeks: slow t + bodyweight - 3~4 weeks slow t + MB 2 KG - 5~6 weeks moderate t + 2 KG - 7~8 weeks fast t + bodyweight	3 sets × 8 reps 3 sets × 8 reps 3 sets × 10 reps 4 sets × 12 reps	
Cool-down (10 min)	Static Stretching			

MB: Medicine ball, CMJ: Countermovement jump, DJ: Depth jump, slow t: Slow tempo, moderate t: Moderate tempo, fast t: Fast tempo.

**Table 3:** SAQ training program.

Time	Type	Intensity	Frequency
Warm-up (10 min)	Dynamic stretching		3 times per week for 8 weeks
Main exercise (30 min)	- High-knees/Butt kicker 3set	25 yds	
	- 1~2 weeks: Wall drill, Arm Action kneeling - 3~4 weeks: Lean fall jog 20 yards, L E F T drill - 5~6 weeks: Figure Eight, M drill - 7~8 weeks: Foot work-2leg, SL 2leg forward, lateral, zigzag, side shuffle, skier, W-wave	2 sets × 6 reps 3 sets × 6 reps 3 sets × 8 reps 3 sets × 8 reps	
	- A, B, Side Skips - Bounding - Carioca - Pro agility - T drill - Ball reaction - Mirroring - Accelerators	1~2 weeks 3 sets × 8 reps 3~4 weeks 3 sets × 8 reps 5~6 weeks 3 sets × 10 reps 7~8 weeks 4 sets × 12 reps	
Cool-down (10 min)	Static Stretching		

**Table 4:** Change in Dynamic balance test (%).

	Group	Before	After 8 Weeks	d		Two-Way ANOVA		$\eta_{p}^{2}$
						*F*	*p*	
CRD-L	NMTG	95.45 ± 7.01	93.81 ± 7.51	−0.23	G	12.858	0.001 **	0.274
	SAQG	101.08 ± 5.65	103.22 ± 5.02	0.4	T	0.119	0.732	0.003
					GxT	7.086	0.120	0.172
CRD-R	NMTG	93.92 ± 7.28	93.92 ± 6.37	0	G	14.218	0.001 **	0.295
	SAQG	100.56 ± 6.14	101.49 ± 2.82	0.2	T	0.319	0.576	0.009
					GxT	0.325	0.572	0.009

Values are means ± SD. NMTG, neuromuscular training group; SAQG, speed, agility, quickness training group; CRD-L, composite reach distance-left; CRD-R, composite reach distance-right; G, group; T, time; GxT, group x time. ** *p* < 0.01.

**Table 5:** Change in Countermovement jump (cm).

	Group	Before	After 8 Weeks	d		Two-Way ANOVA		$\eta_{p}^{2}$
						*F*	*p*	
CMJ	NMTG	47.80 ± 6.36	50.95 ± 6.80	0.48	G	3.79	0.060	0.1
	SAQG	44.33 ± 6.30	46.46 ± 6.41	0.34	T	10.864	0.002 **	0.242
					GxT	0.398	0.532	0.012

Values are means ± SD. NMTG, neuromuscular training group; SAQG, speed, agility, quickness training group. G, group; T, time; GxT, group x time. ** *p* < 0.01.

**Table 6:** Change in Zigzag run test (sec).

	Group	Before	After 8 Weeks	d		Two-Way ANOVA		$\eta_{p}^{2}$
						*F*	*p*	
Zigzag test	NMTG	5.55 ± 0.24	5.59 ± 0.20	0.18	G	2.573	<0.001 ***	0.07
	SAQG	6.04 ± 0.52	5.56 ± 0.75	−0.75	T	14.069	0.001 **	0.293
					GxT	20.268	<0.001 ***	0.373

Values are means ± SD. NMTG, neuromuscular training group; SAQG, speed, agility, quickness training group. G, group; T, time; GxT, group x time. ** *p* < 0.01, *** *p* < 0.001.

**Table 7:** Change in Carioca test (sec).

	Group	Before	After 8 Weeks	d		Two-Way ANOVA		$\eta_{p}^{2}$
						*F*	*p*	
Carioca	NMTG	6.08 ± 0.39	6.13 ± 0.43	0.12	G	1.355	0.252	0.038
	SAQG	6.15 ± 0.74	5.70 ± 0.36	−0.78	T	10.923	0.002 **	0.243
					GxT	18.174	<0.001 ***	0.348

Values are means ± SD. NMTG, neuromuscular training group; SAQG, speed, agility, quickness training group. G, group; T, time; GxT, group x time. ** *p* < 0.01, *** *p* < 0.001.

## Data Availability

The data presented in this study are available on request from the corresponding author. The data are not publicly available due to the protection of personal information.
